# In search of a ‘good number’: knowledge controversy and population estimates in the endgame of hepatitis C elimination

**DOI:** 10.1136/bmjgh-2023-014659

**Published:** 2024-02-27

**Authors:** Tim Rhodes, Kari Lancaster, Sophie Adams

**Affiliations:** 1 London School of Hygiene & Tropical Medicine, London, UK; 2 University of New South Wales, Sydney, New South Wales, Australia; 3 Goldsmiths University of London, London, UK; 4 Deakin University, Burwood, Australia

**Keywords:** Viral hepatitis, Qualitative study

## Abstract

We explore the contentious life of a metric used to assess a country’s progress in relation to global disease elimination targets. Our topic is hepatitis C elimination, and our context is Australia. A fundamental metric in the calculation of progress toward hepatitis C elimination targets, as set by the WHO, is the population prevalence of people living with hepatitis C. In Australia, this modelled estimate has generated some controversy, largely through its repeated downsizing as an effect of calculus. The 2015 baseline population estimate in Australia, from which measures of current elimination progress are assessed, has reduced, over time, by around 30%. Informed by a social study of science approach, we used qualitative interviews with 32 experts to explore the knowledge controversy. The controversy is narrated through the core concerns of ‘scale’ and ‘care’, with narratives aligning differently to imaginaries of ‘science’ and ‘community’. We trace how constitutions of ‘estimate’ and ‘number’ circulate in relation to ‘population’ and ‘people’, and as affective values. We show how enactments of estimates and numbers materialise hepatitis elimination in different ways, with policy implications. The event of the knowledge controversy opens up the social and political life of enumerations—for science and community—inviting deliberation on how to make ‘good numbers’ in the race to eliminate hepatitis C.

**WHAT IS ALREADY KNOWN ON THIS TOPIC**

- Global targets of disease elimination are used to monitor the success of national strategies, as in the case of Australia’s efforts to eliminate hepatitis C, but rarely are the social effects of such measures considered.

**WHAT THIS STUDY ADDS**

- This qualitative study shows that contested estimates and numbers are afforded multiple meanings and values which shape how science and community respond to the elimination of hepatitis C.

**HOW THIS STUDY MIGHT AFFECT RESEARCH, PRACTICE OR POLICY**

- Disease elimination estimates are not ‘just numbers’. When estimates used in national targets change, there is a need to consider the social, material and policy implications, and not only issues of calculus.

## Introduction

The development of numerical targets, and metrics to assess progress about these, is a core feature of intervention and governance in the field of global health.^1^ Global disease elimination targets, like those set by the WHO, shape national strategic efforts and actions, as well as declarations of progress and impact, as is the case in global efforts to eliminate malaria, HIV and hepatitis C.^2–4^ There has been a shift towards indirect estimation, as well as modelling, as measures of progress and accountability in global health more generally.^5^ Rarely are the estimates that are used to monitor progress about disease elimination targets considered in relation to their social effects and values.

In 2016 the WHO set a goal to eliminate viral hepatitis as a major public health threat by 2030.^2^ The first Global Health Sector Strategy on Viral Hepatitis was underpinned by ‘a set of ambitious targets’ including an 80% of the people with hepatitis C treated, an 80% decline in new infections and a 65% decline in liver-related deaths.^2^ The availability of highly-effective direct-acting antiviral (DAA) treatments ushered in a new era in which hepatitis C elimination might be achieved via a treatment-as-prevention strategy and endeavours to ‘find the missing millions’.^6–8^ Despite global efforts to accelerate the elimination response through financing measures, testing and treatment access and simplified models of care, progress has slowed,^9^ and many countries are not projected to meet these targets.^10 11^

Australia has been at the forefront of the global hepatitis C elimination response,^12^ with an initial $A1.2 billion investment in DAA treatments in 2016, and demonstrable early success.^13^ Although once regarded as ‘on track’ to achieve elimination by 2030,^14^ concerns have been raised that progress will stall unless new approaches are implemented in the ‘endgame’ phases of the elimination response.^10 15–17^

Following the endorsement of the Global Health Sector Strategy on Viral Hepatitis, in 2017 the WHO sought to establish global and regional estimates on viral hepatitis in 2015, setting the baseline for tracking progress in implementing the new global strategy.^18^ A prime metric in the calculation of elimination progress, especially in the proportion treated, is the denominator of the target population of people living with chronic hepatitis C. The 2015 baseline population estimate is a critical input shaping elimination projections, resource planning and intervention targeting. Key elements in the estimation of the denominator include cumulative hepatitis C notifications nationally, rates of spontaneous viral clearance, extrapolated population mortality and estimated population migration.^19^

By definition, ‘estimates’ of population prevalence generate latitude. Population prevalence estimates are usually expressed as a numerical range, and qualified in confidence intervals (CIs), given that the absolute number is uncertain or unknown. Modelled estimates generate latitude as an effect of the combinations of their heterogenous inputs as well as how they are iteratively tweaked in relation to observed data altering their parameters and assumptions. Estimates and numbers are afforded further fluidity as they are interpreted and made meaningful in practice and policy.^5 20–22^ The latitude that surrounds estimation and enumeration enables freedom of action or thought in the generation and use of metrics.

It is this *latitude* in the negotiation of ‘estimates’ that we investigate here through a qualitative study of controversy in the numbering of hepatitis C’s elimination. Through a moment of controversy in evidence-making, we trace how a population estimate gets made and re-made, resisted and defended. Our analysis shows how enumerations are afforded fluidity as well as social and affective value beyond claims to relative accuracy. This is important because population estimates are often taken for granted in the setting, implementation and assessment of global disease targets. Once circulated and made official, such numbers—which can have a fundamental impact on policy and practice—can appear concrete and stable, with the latitude of their estimation origins lost from view.

### Population estimates and elimination targets

In Australia, scientists engaged in hepatitis C surveillance and response have reduced the 2015 baseline population estimate of people living with hepatitis C, over time, by around 30%. Published estimates suggested 227 000 people living with hepatitis C in 2015.^14^ But in 2019, this 2015 estimate was revised downwards to 188 690.^15 23^ In 2022, in advance of the Sixth National Hepatitis C Strategy,^24^ scientists proposed a further downward revision of the 2015 baseline to 158 000 people living with hepatitis C. The appearance of dramatic reductions in the baseline—which ostensibly reduced the number of people who are left to treat—has generated some controversy among stakeholders enjoined in Australia’s partnership approach to hepatitis C’s elimination.

Given the controversy, as an interim measure, stakeholders agreed that the current national strategy,^24^ which focuses on the endgame phase of elimination to 2030, should be published for consultation with ‘old’ revised estimates (a 2015 population baseline around 188 000 rather than the proposed 158 000), quoting ‘old’ estimates of people left to treat as the target for elimination (of 117 810 rather than around 80 000). At the time of this qualitative study, current ‘best estimates’ of the number of people living with hepatitis C in Australia were disappearing from view, as well as from policy.

### Knowledge controversy

The focus of this qualitative analysis then, is a ‘knowledge controversy’.^25^ Knowledge controversies are events that make present, as well as deliberate on, the multiple meanings and enactments of evidence in a field.^26 27^ Analyses of knowledge controversy in the science of hepatitis C, for instance, have noted the multiple social and political values afforded by evidence.^28^ Numbers, including elimination targets and projections, have been emphasised as mutable.^20 29 30^ Such is the latitude that shapes the enumeration of global disease elimination targets that it has been suggested that there is a calculative space of ‘virtual elimination’ which performs as ‘anticipatory governance’.^30–32^ There are other examples in global health, such as in the field of maternal health, where the downsizing of estimates has led to controversy, not only over estimation methods and concerns regarding funding and investment, but also over what gets measured and how measures are politically valued.^5^ A controversy over a number, especially one that performs in policy, is an invitation to learn; not necessarily to achieve consensus, and certainly not to close down difference, but to create opportunities to ‘arouse a different awareness of the problems and situations that mobilise us’.^25^ The controversy is an invitation to slow down to reflect on doing things differently,^33^ as we do here in deliberating on what constitutes a ‘good number’ in efforts to evidence and mobilise action in the elimination of hepatitis C.

## Methods

We draw on interview accounts of stakeholders engaged in the hepatitis C elimination response in Australia. These interviews were generated in a consultation proposed by Hepatitis Australia, a national non-profit organisation and charity in Australia representing the interests of people affected by hepatitis C. Qualitative interviews were conducted between mid-March and early April 2023 with 32 individuals working within federal and state governments, universities, non-government and advocacy organisations. Participants’ expertise included the lived experience of hepatitis C, epidemiology, mathematical modelling, disease surveillance, social science, health services research and delivery, global health, policy-making, clinical care, nursing, community-based intervention and community advocacy. All interviews were carried out using Zoom or Teams, with consent, audio-recorded and transcribed verbatim. Interviews aimed to map the diversity of expert perspectives on the methods, practices, meanings, promise and pitfalls of population estimates used in relation to national targets of hepatitis C elimination.

Our analysis aims to bring to the surface the different meanings and values that numbers can afford. We do this by looking at how numbers are made to matter in the interview accounts of stakeholders as well as how numbers are put to use as forms or elements of narrative.^30 34 35^ We coded transcripts initially for emerging descriptive content, with coding further refined in an iterative process of coding, charting and interpretation.^36^ We aimed to trace how accounts ‘enacted’ population estimates, numbers and targets as situated matters of concern.^34^ It is here that our analysis sought to investigate emerging relationships between the themes of ‘enumeration’ and ‘mattering’, as well as distinctions between ‘estimate’ and ‘number’, and how population estimates were afforded different values in relation to ‘science’, ‘community’, ‘scale’ and ‘care’. Importantly, our analysis is not oriented to representing the accuracy of ‘truth claims’ but instead investigates their performance. This means that we are primarily interested in how the accounts of stakeholders situate ideas as well as bring ideas into being.^34^ We acknowledge that we, in our own telling, are co-producers in this story.

In the analysis that follows, we present selected excerpts with the interview participant number in parentheses, but do not present biographical information to reduce the risk of deductive disclosure.^37^

## Findings

We present our analysis about two intersecting themes: *enumerating* (how estimates produce numbers that are afforded latitude); and *mattering* (how estimates and numbers are made meaningful in their situation).

### Enumerating

Our first theme concerns estimations of the denominator of people living with hepatittis C and its revisioning. Scientists accentuate calculation revisions as ‘routine’, ‘ordinary’, ‘business as usual’ and ‘normal’. Revising base estimates is a mark of ‘good science’ which progresses iteratively with the ‘best available evidence’: ‘There is incredible fluidity as new data emerges. […] As time changes and as you get new data, we continue to refine how accurate the estimates are’ (P21). Estimation is here presented as processual. Controversy is attenuated through the claim that revision is in the ‘nature’ of science. We are reminded that ‘epidemiology is dynamic’ and that ‘models are constantly in flux’, with change ‘expected’, as models are ‘matched’ to the ‘real world’, in efforts ‘to get as close as we can to the truth’ (P7; P9; P21). We were told that the best science demands that estimates be revised:

I would be very uncomfortable with any move that said we shouldn’t be approaching the development of these estimates with the most rigorous statistical and epidemiological approach we can, and by revising estimates, that’s actually us doing our jobs as researchers to ensure that we are reflecting our reality in the best possible way we can. (P1)

In this account, there is comfort, rather than controversy, when estimates evolve. Static estimates would signal a problem: ‘If the denominator year in and year out remains static, that’s someone who is not going back to the evidence and trying to find out and improve the model’ (P1). The denominator is presented as ‘realistic’ and ‘right’ but only ‘at the time, when we estimated it’ (P3). Keeping models ‘live’ to the ‘empirical data’ of the ‘real-world’ affords the model ‘validation’ (P3). In seeking such validation, scientists had found that their models were ‘not matching’ the situation as they observed it, especially regarding empirical measures of liver disease (thought to be overestimated) and treatment coverage (thought to be underestimated) in the population of people living with hepatitis C. The denominator was consequently felt by some to be ‘too high’ and an ‘overestimate’:

It was clear to us that the model was spitting out numbers that were significantly higher than the empiric data was telling us. […] So, we looked at the model and thought where could the parameter issues lie. (P9)

On the treatment coverage output, which is a critical viral elimination target, the model was producing estimates in a range that were, according to some, in ‘no way’ correct. Before downsizing the denominator, the model indicated a treatment coverage proportion of around 50% (said to be 48% by some and as low as 36% by others). Whatever the value (and it is quoted as 51% in the latest national strategy^24^), we were told ‘it can’t be less than 50%’; that ‘there is absolutely no way that a minority of people living with hepatitis C since 2015 have been treated, that is just not the case’ (P9).

With the model making ‘clear our notifications were overestimates’, scientists ‘had to alter the parameters’, for a second time since 2019, to ‘adjust’ downwards the baseline denominator. Two input tweaks were made: case notifications; and assumed rates of spontaneous viral clearance. Case notifications of hepatitis C infections in Australia are reported at the state and territory level, which gives rise to the possibility of duplications when the same cases are reported in more than one state or territory. Spontaneous viral clearance is where a positive HCV antibody indicating a history of infection is followed by a negative HCV PCR indicating the absence of virus without treatment. A rate of 9% duplication in notifications across states and territories was assumed since 2019, and since 2022, the rate of spontaneous viral clearance has increased to 36% (from a previously assumed 25%), partly based on estimates elsewhere^38–40^:

We reached a stage where there’s been updated data that has emerged in the field, particularly around the proportion of people that spontaneously clear the infection, and also some issues around how people move between [Australian] states, which have resulted in a lower number of people living with hepatitis C in Australia than perhaps was previously thought (P21)

Downsizing the denominator further in 2022 would increase the treatment coverage proportion to around 56%. Even here though, there was equivocation in interview accounts: ‘If the 56% is too low that means the prevalent population of people living with hepatitis C is too high’ (P9). The increased treatment coverage percentage enabled by downsizing the denominator in the model was going in the right direction, but for some, perhaps not far enough:

It’s much more likely that the number is still an overestimate rather than underestimate. A lot of the angst is around it being potentially an underestimate but I think it’s the other way around. […] Our treatment coverage proportion, based on that reduced number of people living with hepatitis C, is only around 56%. The people that believe the number should be sizeably larger, the corollary of that is they want the treatment coverage to be lower. (P9).

Others also suggested that duplications in notifications might be higher than the 9% assumed, ‘actually somewhere between 10 and 20%’ (P1). Efforts to ‘deduplicate’ make ‘really big impacts’ on the denominator because notification data is historical. Even if the downsizing of the denominator is not big enough for some, there is acknowledgement that the effects of deduplication and higher spontaneous clearance rates are ‘big’ and ‘influential’, producing ‘dramatic’ declines.

#### Estimates afford latitude

Here then, we begin to appreciate how the downsizing of estimates might matter in different ways to different stakeholders (see sections below). For many scientists, the single number is not a matter of concern. Accounts invoke latitude in their defence. Precision about the absolute number, an unknown, is said to be less important than the ‘order of magnitude’ and ‘trajectory’ of change in the target population, and its *relation* to other measures including treatment coverage. The advice is: ‘Do not look at a single number but look at the trajectory of what’s going on’ (P23). When doing so, ‘the magnitude of change is not that great’ (P21). For some, the number ‘makes not a jot of difference’ in practical terms (P23). As commented on the declining estimate of those left to treat:

There is a bit of latitude. I really don’t think things would have been very different whether it’s 100 000 or 80 000, whether the treatment uptake was 48% or 56%, it’s still a huge number of people, large proportion of people, that need to be reached in terms of the strategic goals and programmes. (P9)

If somebody says, ‘You’ve got 70 000 to treat or 85 000 left to treat’, I go ‘How does that change what I actually have to structurally do in my thinking and in my work?’ (P23)

In this account, the precision of the number per se ‘does not actually matter’. The latitude that surrounds the *estimate* does not alter ‘the reality that Australia is not on track’:

In some ways it doesn’t actually matter if the number goes down by 30%, there’s still a lot of people living with hep C who still need to be cured. (P26)

Even if this change places us closer to elimination, the point is we’re still not going to make it in time. […] The change in number won’t change government response materially. […] If I’ve got 70 000 people left to treat in Australia or 90 000 people left to treat in Australia or 120 000 people left to treat in Australia, okay, it's still tens of thousands. (P23)

The accounts of scientists emphasise the need ‘to get this number correct so that we can measure progress against the elimination targets’ as well as to ‘match’ it as ‘accurately as possible’ to the ‘real-world’ (P21; P9; P3), but at the same time, they accentuate the latitude and fluidity of estimation to caution against unrealistic assumptions of numerical stability and precision. The latitude in estimation is such that one scientist notes that while downsizing the estimate might be the ‘best thing to do’ it is ‘a brave thing’, because ‘we have no idea how accurate [estimates] are in how they represent the truth of the number of people living with hepatitis C, no one knows’ (P18). Users of the estimates (eg, those working in policy, advocacy and practice), some scientists suggest, ‘potentially do not understand that things do change, and that we need to be prepared for it’ (P21). Taken together, the denominator is said to have been afforded ‘too much power’, as if ‘Gospel’, appearing *too much as a number*, and *not enough as an estimate*:

There’s a bit of an own goal that’s happened over time where the original numbers have been considered to be as Gospel and, you know, really this is the number, when we should’ve always been talking about it as an estimate, and making it clear that it was just an estimate (P11)

The controversy over downsizing the estimate is here shifted as a problem of the perception that ‘the number is the number’. An appeal is made to the ‘fine print’, the ‘caveats’, that scientists say they communicate about the uncertainties of estimates to indicate that *numbers should not be treated as such*:

People have this view of the number is the number. Well, actually it’s not. The number is as best as at the moment we can work out. We always write caveats around it, if you read the fine print, but as most people don’t bother to read the fine print, they don’t go, ‘Oh, there’s caveats around here, and those confidence intervals are there for a reason’. But most people don’t know what confidence intervals really are. (P23)

### Mattering

The changing estimate of the number of people living with hepatitis C is made to matter in multiple ways. Matters of ‘scale’ and ‘care’ are core concerns. These concerns are situated in relation to different constitutions of ‘estimate’ and ‘number’: first, as an index of viral elimination progress against population targets in time; and second, of people treated and left to treat. The first configuration leans towards an epidemiological population-based imaginary, which presents as a concern of calculus and projection, whereas the second accentuates an affective attachment and community of engagement actualised in the present, in people and care experiences. Latitude seems more acceptable in the first but less so, and even sometimes violating, in the second. These different versions of estimate and number are mapped in accounts to imaginaries of ‘science’ and ‘community’. Accounts of the estimate as a number that translates to ‘actual people’, and which therefore also acts as ‘more than just a number’, are aligned primarily with people living with hepatitis C and people who advocate on their behalf, and less so with surveillance and science.

#### Narratives of scale

Our analysis considers the number as a matter of narrative (how the story is made), and not simply calculus (how the calculation is made). The denominator ‘can be shifted in direction depending on the narrative that is around’ (P5). Whereas altering the estimate can be minimised as ‘ordinary’ and ‘mundane’, and within a latitude that does not present cause for concern, as noted above, it can also be narrated otherwise, as ‘big stuff’. For instance, in contrast to the accounts of some scientists, the accounts of some community stakeholders emphasised: ‘This isn’t a usual ticking over of the numbers, this is a major, this is a major change that’s proposed’ (P28). The controversy situates altered estimation as a site of ‘scale politics’, producing ‘scalar narratives’^40^ in the sizing and shaping of hepatitis C as a matter of policy and community concern. Scalar narratives are those that use scale as a resource to explain events and present an argument,^41 42^ here about hepatitis C. Here is one perspective on this deliberation:

I think what do we want this number to be is a tricky one, because what we want this number to be from the Department of Health is we want it to be small because it’s a low burden of disease. But what we want this number to be from resourcing of other areas might be that you want it to be bigger because that’s then going to ensure resourcing and allocation of staffing and things like that (P5)

In scalar narratives of elimination progress, the population estimate is prime. It is the ‘primary number that drives the response’ (P28). It ‘defines scale’ (P16). It is seen as the base from which ‘everything flow(s)’ when measuring testing, treatment and other targets. The number acts as a ‘proxy indicator of urgency’ (P12) and of ‘need for action’ (P4), shaping high-level policy (P10; P12) as well as the on-the-ground work plans of service providers (P7; P28; P29; P31). The number is circulated ‘everywhere’, in ‘every report’, and ‘gets used at every single thing’ (P28). Whatever the latitude of its estimation, the effects of this number are ‘real’:

Areas of action, and priority populations, and priority settings, within the strategies are sort of built around that number essentially, and if the number changes and that sort of gives rise to the downstream effects. (P10)

From a national number, it flows down like to people’s individual workplans. It is full-on how much this number is real. (P28)

This stuff matters. This is not mathematics published in a report somewhere that gathers dust, this modelling makes national policy. (P12)

Experience suggests that the number is ‘shockingly powerful’ and can make a material difference:

It’s been quite powerful for us in our advocacy to be talking about targets that had been missed. (P4)

The metrics-driven focus on progress and success is what perpetuates the yearning to get the number as ‘right’ and as ‘realistic’ as possible. There is said to be something of an ‘obsession’ with the base numbers that link to targets in the race to eliminate:

I work really closely with policymakers, including drafting things like the national strategies, and what I’ve noticed working with policymakers in this space is they’re quite obsessed with these numbers. (P4)

It’s important to understand the burden of disease in the population, and it’s important to get this number correct, so that we can measure our progress against the elimination targets. (P21)

As Australia enters the ‘endgame’ of hepatitis C elimination, scaling the problem intensifies. Measures of base population prevalence have come to matter more, and matter differently, since the arrival of DAA treatments, and with elimination becoming a probability, not mere possibility.^13^ Yet Australia’s progress against global elimination targets has ‘slowed’, moving from ‘fast-track’ and ‘early’ elimination to ‘missed’ targets and to no longer being ‘on track’.^14 15^ These shifts situate the controversy: ‘All of a sudden the number matters in a different way’ (P26); ‘The reason this [estimate] has such power is particularly the point that we’re at in Australia’ (P5). There is a ‘need to know’ (P13; P5):

We need to know […] if we’re getting close to or near endgame elimination. We know we have to try different approaches the closer we get to […] endgame elimination. (P20)

The concern here is how altered estimates reshape ‘policy narrative’, including bringing Australia back ‘on track’. A reducing base population may help to re-enact a scalar narrative of progress being made: ‘Our concern was like a major shift in the policy narrative from not being on track to kind of being on track’ (P4); ‘You change that number and you can model your way out of the problem’ (P28). It ‘suits the agenda now, for the estimate to be low’ (P12). We see contrast then, in how scalar narratives are imagined to align with imagined networks of expertise, with science and policy actors presumed more comfortable with a reducing population estimate than community actors concerned that this might undermine hepatitis C represented ‘big’ and ‘big enough’ going forwards. Any lessening of policy commitment is feared to reverse progress made (P16). A lesser target population ‘implies the problem is not as big as it is’ (P25). It creates ‘policy ambiguity’ (P4). In the endgame, uncertainty is unhelpful, because ‘the same, if not more resourcing is required’ (P7). Because ‘it actually gets harder and harder to achieve the same gains that you’ve seen previously […] it actually needs an increase in resources’ (P2). The endgame scalar narrative needs to perform things ‘big enough’ to maintain momentum: ‘This is a moment where you should hold your form’ (P23). There is, therefore, also a nuanced distinction in ‘scale politics’^37^ between the size of the *population* in need and the size of the *problem* at hand, with the reduction of the former indicating greater, if not the same, resource need going forward.

The event of controversy over population estimation can thus be seen as a deliberation on how *big* the matter of concern is represented to be, and what downsizing the population of people living with hepatitis C *does* to the narrative *now*:

We really need to be investing here. We need to be scaling-up. Now is the time in the epidemic. We’ve seven years left to achieve elimination, and a concern, not looking at the numbers in detail yet, but just knowing that it had been revised down, immediately was, ‘Shit! Well, what if this makes the narrative now?’. (P4)

This question of ‘what makes the narrative now?’ accentuates the potentials, and not just the pitfalls, of enumeration as an ‘evidence-making’ event^35^; that is, as a site not only of controversy but of opportunity.^22^ What potentials then, might different versions of numbers afford? Here, accounts emphasise how the downsizing of the target population intensifies the pressure to justify investment at a critical point in Australia’s elimination challenge, with the potential to ‘change the discourse’ in policy, planning and priorities:

Unfortunately, in government, those conversations can get pretty kind of simplistic and reductionist. So, where you’ve got clear evidence that a problem is big and expensive, it’s sort of easier to go and have arguments as to why investment should follow. I don’t think that necessarily leads to a scenario where if the number is revised down because of, you know, newer surveying and resourcing and updated modelling, that money goes away, but it does mean that you have to better articulate why […] maintaining investment to achieve that is such an important goal, and that what you’re investing in is going to get you there. So, in a situation where if the numbers drop substantially, […] I think it just changes the conversation in a way that people start to go, well, how much investment do you need? Why are you investing in it? It requires greater clarity.’ (P8)

#### Narratives of care

Narratives of scale intersect with those of care. The downsizing of the population estimate is accounted as a *lessening* which is not merely felt in numerical terms but as a form of devaluing and discounting in other ways too:

For people living with hepatitis C, all of a sudden it’s not as a big a problem, which for some people is a good thing, but for other people potentially minimises what they are experiencing. […] This has been done and all of a sudden there is less people. […] We’ve just changed the number of people overnight. As an individual, it feels like you’ve either just been taken out and tossed aside, or you know, put back in. (P5)

In this narrative, there are *fewer people*, and people who matter have gone ‘missing’: ‘40 000 people seemed to disappear from the prevalence number overnight, and there has been some questions raised about where did those people go’ (P5). This is the narrative that envisages the estimate as an absolute, as a *number* that represents actual individuals. In this articulation, the number is not a mere estimate, and moreover, it is *more-than* a number. When translated as not ‘just a number’, ‘It is people that we’re talking about, the numbers reflect people’ (P16). ‘People see this as people, they see it as themselves’ (P28). This is why getting the number ‘right’, ‘accurate’, ‘correct’ and ‘true’ is also a concern of those working face-to-face with affected people (P20). In this narrative, latitude and fluidity in estimation are part of the problem and cause of the controversy rather than part of the defence which seeks to moderate it. Because ‘the number really matters’ in representing lived experience—of people, care and treatment—the claim that estimates afford numbers a wide latitude that ‘does not matter’ in practice is contested. The contestation here then, is the navigation between an *estimate* that is also a *number*: The changing number is ‘not really real, but real’ at the same time. For instance, it is not that there is a misunderstanding that estimates are ‘the result of an equation’ that do not necessarily represent ‘individual people’—the science is relatively well understood—but that this is how they are *felt* in experiential terms. As commented:

I am a consumer of the numbers. […] So the numbers actually mean something to me. […] I’ve learnt recently that the numbers perhaps are not individual people. I think of them that there are 117 000 people with hepatitis C in Australia. I think of them as individuals and people (P7)

The downsizing of the denominator is thus materialised in these accounts as people that were once there but are now ‘never there to begin with’. While this downsizing in science-based narratives does not fundamentally alter the ‘reality’ of ‘not being on track’ and of what therefore needs to be done (see above), it is *ontologically unsettling* for some: ‘If the number is not real’, then what is? This is a lessening that is not merely numerical but translated as an absenting and disruption of *experience*, including in relation to care and capacity for action. Calculus translates as *affective value*, with ‘lost people’ no longer ‘there’ troubling the ‘real’ of care invested as well as projected. The number is not merely ‘the size the problem, it’s the justification for the work’; a ‘measure of the work that has been achieved’, and ‘the worthiness of the investment’ that, taken together, constitutes *care*:

They are out there doing stuff, and it’s hard work, and it’s frustrating work. […] We’ve treated 100 000 people, it’s something that we’re really proud of, and we should be bloody proud of it. Yeah, so the anger that I heard last year when suddenly 40 000 were just dropped off overnight. It was like, well, we don’t have to do anything from now on, because the number will just keep dropping, and we’ll reach elimination targets by 2030. It overlooks the effort. (P7)

The ‘big impact’ of altered estimates is recognised among some scientists, who reflect that they may ‘underestimate’ the social effects of how numbers become materialised in experience:

If I’m a person living with hepatitis C or if I’m an advocate who has been working in hepatitis C, I see the number of people living with hepatitis C drop from an estimated 120 000 down to 80 000 in one fell swoop. (P1)

We’ve got 30 000 less people now, so instead of 110 000, you’re saying there is only 80 000 people. Like, how can that be? Like, how can you just drop off a third of the population that we think has hepatitis C? (P9)

The ‘sudden’ loss of people is here positioned, by some, as ‘care-*less*’, representing a discounting of the care effort. The altered number presents as a ‘bombshell’ that is ‘blindsiding’, as not careful enough, and as a reduction that is ‘undermining’ the strategic response, as care-less. Not only is the timing of the downsizing in the number of people living with hepatitis C made contentious because it coincides with the launch of national endgame strategy, but it was said, by some, to have happened ‘overnight’ as a ‘surprise’ (P25), and presented as a ‘done deal’ (P4). The revision was likened to ‘shifting the carpet under us’ (P1). Coming too fast, the downsized estimate generates a sense of unease and lack of preparedness which in practical terms is not without risk. Concerns here included damage to reputation and credibility, for instance, in upending the capacity of stakeholders to have a narrative of confidence when accounting for a lesser population:

80 000 or 70 000 people no longer exist in that figure and that’s […] quite a significant thing to explain to people. (P16)

The ‘care-less’ effects of downsizing the denominator thus combine a sense of discounting across people, care experience and community-science relations. Here, the controversy is also enacted as a matter of *respect* and *trust*:

A disrespect for the rest of the sector, I think, that this is an important number, that we’re all in this together, and we’ve all got to understand where the number comes from. (P7)

Nobody’s disputing science changes or things change or anything like that, but you can't get around three times in 3 years, and change the number that significantly, and then stand there with a straight face to the community and be like, ‘we've got it’. (P28)

I think that the most unsettling part would be that there was just such a dramatic reduction in the numbers, and what does that say about the credibility of epidemiology itself? (P31)

With ‘community’ attributed ‘deep emotional attachment to these numbers’ because they ‘reflect people’—people in need, people cared, people cured—accounts appear to be in search of what constitutes a ‘good number’. A ‘good number’ here merges ideas of calculus (a good enough scientific estimate of people and population) with ideas of what numbers embody in their social meanings and values (measures that reflect actualised experience and capacity for action) with ideas of *numbers that care* (measures that are valued as fair and just). These versions of numbers each materialise or seek correspondence with reality, but they do so differently, and multiply at the same time. This emergent notion of ‘good number’ in its situation suggests an emergent *ethics of enumeration* for the field. Here is an account that begins to traverse some of these multiple versions of ‘good number’, in the face of unsettled community-science relations:

When the numbers change, you know, people want the numbers to change in a really *good way*. Because it’s them and it’s their families and it’s their people and they like want them to be *good numbers*. Now, nobody wants a bad, they don’t want bad numbers, they don’t want numbers to stay bad, their interest isn’t like ‘let’s keep people sick’ or anything like that. But they want people to be treated *fair*, and to be treated *right*, and to be done *justice* in the response. And they want to *trust* the thing. And I think part of this change process, it’s diminished the trust between the community and the numbers. (P28; emphasis added)

## Discussion

Metrics and targets are forms of governance.^1 43 44^ Estimates about global health targets are indicators of progress that help justify programme investments as well as shape political priorities.^1 5^ In the race to eliminate hepatitis C globally by 2030, there is methodological attention given to how measures of viral elimination perform,^45–47^ but rarely do we ask how such measures perform socially and materially.^20^ This invites a different mode of questioning, no less, and perhaps more, important, which is: what multiple meaning, value and impact do measures have across the domains of science, community and policy?

Afforded by the event of a knowledge controversy over baseline population estimates used in national surveillance and policy in the elimination of hepatitis C in Australia, we have explored the contentious life of a metric to surface its multiple meanings, values and impacts. This metric—which enumerates the number of people living with hepatitis C—is *more than calculus*. While science-based accounts give primacy to methodological concerns in calculating epidemiological estimates, there are multiple alternative constitutions of ‘estimate’ and ‘number’ at play. This is a reminder that calculus is ‘nothing’ without its social relations, and calculations are never without affective or political value.^48 49^ The controversy over population estimation in Australia is a deliberation in how numbers are afforded competing epistemic and affective value with implications for care. Numbers are never ‘simply numbers’ that ‘speak for themselves’.

### Estimate and number

We found that contrasting narratives of ‘scale’ and ‘care’ were aligned differently in relation to the imaginaries of ‘science’ and ‘community’. This is how the event of the controversy was performed in the narrative to enact value differently—in numerical, material and affective terms—regarding the problem of hepatitis C and its elimination. We emphasise the figures of science and community as *imaginaries*; that is, enactments that emerge in narratives deployed to signal competing accounts of the controversial situation. We accept that these depictions are not clear cut and intersecting, and that they coordinate within them varieties of expertise and experience. Whereas science-based accounts of estimation minimise connotations of controversy by emphasising latitude wherein epidemiological estimates are presented as ‘less-than’ numbers, and perhaps not even numbers at all, enactments of numbers in community-based accounts make estimates contentious because they signal ‘more-than’ estimates, and furthermore, ‘not just numbers’.

Science-based accounts tended to constitute *estimates* as distinct from numbers, also affording them latitude; a scope for freedom of action or thought. The controversy is problematised here as a slippage of the estimate to number. Considered as modelled estimates, iterative revision and latitude, is acceptable; in fact, a signifier of good science, which is perhaps, sometimes, misunderstood. This account reads the estimate in relation to a population-based imaginary, that is, as an indicator of national-level population change in an epidemiological trajectory of progression towards a virtual target. Crucially, the estimated reduction in the base population—in the order of 30%—is not considered a matter of concern, since although appearing ‘big’, it does not change the epidemiological ‘reality’ that there are many thousands left to treat and global targets are still being missed. Although the incremental downsizing of the 2015 baseline population estimate suggests marginally better viral elimination progress than was thought a few years ago, this account claims correspondence to the ‘real-world’ while resisting the suggestion that reducing the target population will make a material difference to investment or policy. The epistemic claim of this account resides in epidemiological, modelling and surveillance expertise.

In contrast, community-based accounts tended to envisage estimates as *numbers*, representative of ‘actual people’, and valued as a measure of care and affective engagement. Here, latitude surrounding the lessening of the target population unsettles the experience of the embodied ‘real’, and can be felt as a form of discounting or devaluing, limiting capacity for action. In this account, estimates are not only materialised as numbers, imagined in the realm of people and embodied in lived experience, but they are also performed as not ‘just numbers’ to signal that they are afforded social and material life beyond calculus. Estimates then, are ‘more-than’ numbers because they *do count* and *matter*. This is because they hold affective value as a measure of the materiality of care and because the order of magnitude of reduction in the estimated number is felt to be ‘big’ and ‘big enough’ to potentiate material changes in future investment. The downsizing of the population estimate is here enacted as *ontologically unsettling* for, unlike the science-based account above, it alters the sense of the ‘real’, both removing people and experiences that had been felt to exist, and creating an elimination future unsettled. Taken together, this account accentuates the lessening of people through altered estimation as ‘care-*less*’ in its apparent discounting of people, lived experience and care engagement. The epistemic claim of this account resides in the lived experiences of hepatitis C, care and advocacy.

### Numbering differently

In surfacing estimates and numbers as carrying social and material values which align differently in networks of ‘science’ and ‘community’, it becomes possible to appreciate how that which might appear ‘mundane’ and ‘ordinary’ can also ‘really matter’. At the time of writing, there is a ‘pause’ on the use of the latest (2022) downsized population estimate in the national hepatitis C elimination strategy. Such is the controversy, that the latest estimates of the number of people living with hepatitis C are disappearing from view, at least for now. This is not a question of which single estimate or number is ‘more real’ than the other. This is not a question of making a simple choice between calculations and affects, or of finding consensus in a singular calculative or affective space.^48^ Contrasting narratives of estimation and numbering correspond to multiple enactments of the ‘real’ of the endgame of hepatitis C’s elimination—whether these be the epistemological logics and claims of science or advocacy, the lived experiences of people engaged in the field, or the intersections that arise across these.

The event of controversy opens up the potentials for narrating and numbering differently. First, our analysis accentuates narrative over number. Here, there is an opportunity, as productively recognised by stakeholders, to focus attention on the strategic narrative of the endgame, in which numbers, of different kinds, play a supporting role. Second, our analysis suggests destabilising the population estimate as a prime and singular metric. Quite apart from how stable this particular estimate or number might be held to be, it is one of many measures, with the potential also to develop new sets of enumerations going forward, and, indeed, targets of different kinds which might afford different imagined futures. The endgame of elimination calls for a variety of measures beyond those of population, testing, treatment, cure and mortality to also encompass service delivery, care engagement, quality of care experience and the social-systemic factors shaping these, and not only in people ‘left to treat’ but among those treated who remain engaged in care.^50^ Furthermore, we see an emerging interest in deliberating on what constitutes ‘good numbers’ for the field. This suggests an emerging ‘ethics of enumeration’ by holding together multiple versions and values of numbers to enable *numbers that care*; that is, enumerations that are valued as fair and just with health improvement potential.

## Data Availability

All data relevant to the study are included in the article or uploaded as supplementary information.
